# Two Sides of the Same Coin: The Relationship Between Modern Racism and Rape Blaming Attitudes among Swedish Students and Community Members

**DOI:** 10.1177/0033294120978158

**Published:** 2020-12-13

**Authors:** Mattias Sjöberg, Farhan Sarwar

**Affiliations:** Department of Psychology, 4396 Lancaster University, Lancaster, UK; Department of Police Education, Södertörn University, Stockholm, Sweden

**Keywords:** Modern racism, rape victim blame, rape perpetrator blame, rape perception, community members

## Abstract

The objective of this study is to investigate the relationship between modern racism and rape victim and perpetrator blame, and rape perception. Participants from both a community population (*n* = 211) and a student population (*n* = 200) read a rape vignette and provided their judgements of blame towards a victim and perpetrator, their perception of the event as rape, and later answered the modern racism scale. Results showed a significant *positive* relationship between modern racism and rape victim blame (*r* = .35, *R*^2^ × 100 *=* 12.1%), while modern racism had a significant *negative* relationship with perpetrator blame (*r* = −.27, *R^2^* × 100 = 7.5%) and rape perception (*r* = −.29, *R^2^* × 100 = 8.7%). Implications for the criminal justice system as well as suggestions for future research were discussed.

## Introduction

Both racism and sexism have been studied extensively and linked to selective encoding and confirmation biases that uphold stereotypes about women and African Americans (Bulmer & Solomos, 1999; Fiske & Taylor, 2013; Glick & Fiske, 1996; Swim et al., 1995). Previous research suggests that racism and sexism are related (Aghasaleh, 2018; Bäckström & Björklund, 2007; Bergh et al., 2016), and may even be explained by similar underlying constructs, such as the social dominance orientation, empathy, or right-wing authoritarianism (Bäckström & Björklund, 2007), indicating that racism and sexism, as well as other types of prejudices, share fundamental properties. It could be argued that a subtle form of sexism might be rape victim blaming attitudes, which have been studied in several previous studies (e.g., Canto et al., 2018; Donovan, 2007; George & Martinez, 2002; Katz et al., 2017; Sjöberg & Sarwar, 2020; Strömwall et al., 2014). Specifically, when asked to evaluate an ambiguous rape situation, it could be hypothesized that individuals high in racism would be more prone to blame a rape victim and exculpate the perpetrator compared to individuals low in racism. These results have been demonstrated in earlier research among university students (George & Martinez, 2002; Katz et al., 2017). However, few studies have investigated if this relationship is also present in samples of community members. Moreover, there are no studies (according to the authors’ knowledge) that have investigated the relationship between modern racism and rape victim and perpetrator blame from a Northern European perspective. Therefore, this study fills a salient research gap by investigating the relationship between modern racism and rape victim and perpetrator blame, and rape perception in a Northern European context; specifically, among students and community members in Sweden.

In light of the recent racially motivated assaults on African American individuals across North America (Gibson et al., 2020), it is increasingly important to understand the relationship between modern racism and other types of prejudicial behaviors, such as excessive victim blaming and secondary victimization of rape victims (Orth, 2002). This knowledge would hopefully inform future decision makers on how to ensure a just and fair treatment for rape victims in the criminal justice system.

## Modern racism

Racism and xenophobia were generally normalized among the colonizing nations as early as the 17^th^ and 18^th^ centuries (Curtin, 1999), and thus have been around for a significant period. While the history of racism is long and extensive, some researchers have suggested that it has somewhat changed in nature during the last several decades. Specifically, from old fashioned and blatant racism to more covert and modern forms of racism (Levchak, 2018). Whereas the older forms of racism highlighted biological inferiority among non-White people, newer forms of racism (such as modern and symbolic racism) instead focused on African Americans’ perceived undeserved advantages in society (e.g., affirmative action programs) and the endorsement of general conservative attitudes (Henry & Sears, 2003). To measure modern racism more objectively, McConahay (1983, 1986) developed the modern racism scale. One of his studies looked at hiring decisions and how they were influenced by the job applicant’s race (together with their level of modern racism). He found that, among participants with high modern racism, modern racism had a *positive* relationship with the hiring decision of an African American job applicant when the social context was favorable (he had a good résumé and GPA), but a *negative* relationship when he did not exhibit those same positive qualities (McConahay, 1983). This highlights the ambivalence many minorities experience; when they are high performing, they are celebrated by the majority community as exceptional individuals, while once they commit a transgression, they, and the social group to which they belong, are judged very harshly (Van Laer & Janssens, 2011). A recent example of this tendency was shown among successful African American NFL quarterbacks in American football, who were judged more harshly when excessively celebrating after scoring a goal compared to their White counterparts (Corbin & Burrow, 2020). Supposedly, the unduly celebration might have created a “hubris penalty” that led to excessive punishment against the African American quarterbacks.

Modern racism has also been demonstrated to be related to juror decision making outcomes. Specifically, in a hate crime situation, when the victim was a Black man, modern racism was related to the sentencing decision and perpetrator blame among a sample of mock jurors (Gamblin et al., 2018). Interestingly, homosexual prejudice was not related to the sentencing decision of a gay man which indicates that racial prejudice (and modern racism in particular) may be more influential than homophobic prejudice for affecting sentencing decisions of perpetrators.

The Swedish version of the modern racism scale was translated by Akrami et al. (2000), and has since been used in several recent research studies (e.g., Bergh et al., 2010; Bergh & Akrami, 2016; Lindström et al., 2014). Importantly Sweden has gone from a very homogenous country to a multicultural country in just a few generations (Runblom, 1994). Therefore, race is not a widely used concept to describe individual differences in Sweden or the rest of Scandinavia (Bitsch, 2019; Martens, 1997). Instead, the word immigrant is often used to describe someone from a non-European background, and more specifically, from the Middle East (BöRtz, 2007). One reason for this could be that a large proportion of the immigrants in Sweden have come from the Middle East (Statistics Sweden, 2010). Therefore, in the current study, the words immigrant/native can be thought to somewhat resemble the Black (minority)/White (majority) division that exists in North America (Bitsch, 2019).

In line with this reasoning, research has suggested that attitudes toward immigrants and stigmatized racial groups share many similarities (Hooghe & Dassonneville, 2018). In Sweden in particular, attitudes toward immigrants have been used by researchers to capture subtle and old fashion forms of racism (Akrami et al., 2000), indicating the similarities between attitudes toward immigrants and stigmatized racial groups. With the rise of immigration, research has shown that although Swedes have become more accepting of interracial intimate relationships, many still believe these not to be accepted by the majority society (Osanami-Törngren, 2011), similar to the situation in North America (Field et al., 2013). Furthermore, evidence suggests that Swedish adoptees from non-European countries experience subtle forms of racism in their everyday life (Hübinette & Tigervall, 2009). As such, it could be expected that the current results would resemble previous North American studies such as Aosved and Long (2006), George and Martinez (2002), and Katz et al. (2017) who found significant relationships between racism and rape myth acceptance or rape blaming attitudes, as well as other studies which have found generally positive relationships between racist and sexist attitudes (Bergh et al., 2016). Hence, we hypothesized that there would be a positive relationship between modern racism and rape victim blame and a negative relationship between modern racism and rape perpetrator blame.

## Modern racism and rape victim blame

As mentioned previously, several researchers have suggested that racism and sexism are related (Bergh et al., 2016; Glick & Fiske, 1996; Lewis, 1977; Swim et al., 1995). While there are differences in the social relationship between men and women compared to White and minority members (the most obvious being that men/women often rely on men/women for sexual and intimate relationships; Glick & Fiske, 1996), there are also similarities in how one powerful social group (men and/or majority members) relate to another less powerful social group (women and/or minority members). As a result of this, one could theorize that racism (in the form of modern racism) and sexism (in the form of rape victim blame), would be related.

For instance, a study by George and Martinez (2002) demonstrated that higher modern racism scores among participants were associated with seeing a rape victim as more culpable and less credible and giving less responsibility for the event to the perpetrator. Interestingly, while the influence of men’s racism on victim blame was independent of the race of the victim or the perpetrator, women’s racism scores showed a different pattern. Women tended to blame interracial victims (White victim/Black Perpetrator or Black victim/White perpetrator) more than intraracial victims (White victim/White perpetrator or Black victim/Black perpetrator). One could speculate that one reason for this might be the perceived inappropriateness of women to affiliate with people from different ethnic backgrounds.

More recently, Katz et al. (2017) manipulated the name of a potential rape victim in a high-risk for sexual assault situation to appear stereotypically Black or White. They then asked White women of different levels of symbolic racism (similar to modern racism) to assign blame to the victim. It was demonstrated that women who scored higher on symbolic racism assigned higher blame to the potential victim, but that there was no interaction between the victim’s race and the participants’ level of symbolic racism. A follow up study using the same high-risk sexual assault situation found that victim blame was attenuated by emphatic concerns about racial inequality (Katz et al., 2018). These studies suggest that symbolic and modern racism could be related to rape victim blame, but that high awareness of racial injustice may sometimes reduce adverse victim blaming of stigmatized racial groups. Further research has confirmed the links between racist attitudes and rape myth acceptance and negative attitudes toward the rape victim (Mulliken, 2005). In sum, this indicates that modern racism may be able to explain some of the differences in rape victim blame and might be an important variable to consider when trying to combat the detrimental effects of rape victim blame in the legal system, such as secondary victimization (Orth, 2002).

Recent research has also demonstrated that sexism (in the form of protective paternalism) was related to modern racism, less support for the Black lives matter movement, and a greater belief that a police officer had acted appropriately in the killing of an African American man (McMahon & Kahn, 2018). Interestingly, experimentally inducing social threat resulted in more protective paternalism among the men which, in turn, was related to their racial attitudes, whereas no such relationship was observed among female participants. This again suggests that racism and different forms of sexism (e.g., protective paternalism) may be related and that this relationship might be stronger in men, especially under conditions of social threat.

## Perpetrator blame and racism

Naturally, while most studies in the rape blaming literature have focused on victims, some studies have also looked more closely at people’s perceptions of the perpetrators. For example, Bongiorno et al. (2016) described perpetrators in an Australian rape case as either coming from a similar (England or America) or foreign (India or Pakistan) cultural background and the behavior of the victim as either resistant (stereotypic victim behavior) or non-resistant (counter-stereotypic victim behavior). They found that when the victim acted in a counter-stereotypical way, participants blamed the victim more and the perpetrator less, but only when the perpetrator had a foreign background. This suggests that the victim who affiliated with a foreign man potentially was penalized for that behavior, by being blamed more (and by blaming the perpetrator less), but only when the victim did not actively resist the attack.

Another study focusing on majority and minority rape perpetrators in Norway found that minority perpetrators accused of rape tended to be stigmatized and shamed by legal jurors (Bitsch, 2019). In contrast, for majority perpetrators, guilt would constitute the dominant moral sentiment. Importantly, whereas notions of guilt merely focus on the wrongfulness of the action, shame centers in on the perpetrator’s character, which is believed to be more firm and unalterable (Cohen et al., 2011). The difference is that guilt is related to rehabilitation and reintegration whereas shame deems a person’s character to be flawed and makes reintegration into society more difficult (Braithwaite, 1989). Hence, the minority perpetrators could be argued to have been more blamed and condemned for the event.

However, although perpetrators from minority and African American backgrounds often are blamed more in cases of rape, recent research has shown that in certain instances, such as when an accused rape perpetrator was a successful athlete, the public discourse might sometimes change in the perpetrator’s favor (Ash et al., 2017). Specifically, when analyzing tweets related to the decision to not charge a successful African American athlete with rape, it was found that although many tweets were critical of the decision not to charge the perpetrator, the majority expressed support for the decision. Hence, some racially marginalized perpetrators might be able to (if particularly successful) transcend their race and activate more traditional and stereotypical notions of myths about rape which might tend to put more blame on the victim (Ash et al., 2017).

## Authoritarianism, social dominance orientation, and feelings of ambivalence

As mentioned above, both racism and sexism could potentially be related to other underlying constructs. For example, Adorno et al. (1950) argued that there was such a thing as an authoritarian personality that predisposed people to dislike several minority groups (e.g., Jewish people & Black people), and to have conservative political opinions (e.g., women should not marry someone from a different race). Later, Sidanius and Pratto (1999) tried to synthesize these different theories about racism and sexism into one coherent theoretical framework. They came up with the social dominance theory, which postulated that most societies are organized around hierarchies with some groups or people having more power than other groups or people. In other words, one widespread part about human nature might be our desire for organizing structures and hierarchies among our societies and individuals.

Interestingly, social dominance orientation has been found to be related to rape victim blaming. For instance, a recent study found that participants higher in social dominance orientation assigned more blame to a rape victim, and that this effect was stronger when the perpetrator came from a lower socioeconomic background (Canto et al., 2018). One explanation behind these results could be that participants high in social dominance orientation may view women who affiliate with members of a stereotyped group (e.g., a person from a low socioeconomic class) as behaving against traditional female norms and hence more deserving of encroachment. Although the current study did not measure participants’ social dominance orientation or right-wing authoritarianism per se, these concepts could offer an overarching theoretical lens in which to interpret the current findings and connect it to the wider research literature.

Another interesting connection between both racism and sexism relates to their association with feelings of ambivalence (holding both seemingly positive and negative attitudes about marginalized groups such as women and African Americans; Glick & Fiske, 1996; McConahay, 1986 ). Specifically, in relation to feelings of ambivalence, a need among some individuals to be perceived as egalitarian and unprejudiced can sometimes be conflicted with hostile attitudes toward women and/or minority members (Aronson, 2012). Thus, the protagonist will only display their true feelings about women or the minority group when the situation is ambiguous, and it is hard to separate individual prejudice from other more objective reasons for a particular decision. An example of such a situation might be an ambiguous sexual encounter where it sometimes could be possible to find exonerating circumstances for the perpetrator’s behavior, which then may leave more blame on the victim. Therefore, based on this, it could be hypothesized that people with higher levels of modern racism would put more blame on the victim and less blame on the perpetrator in an ambiguous sexual encounter (such as in this study).

## Value of the present study

We believe that there is a paucity of research investigating the relationship between racism and rape victim and perpetrator blame, especially from a non- North American perspective. Since it is well known that North American culture (e.g., United States) differ from many other cultures, such as Sweden (Hofstede, 1991; Maud, 2016), this research is warranted and relevant. Furthermore, Sweden is considered to be one of the most egalitarian countries in the world (World Economic Forum, 2018), and therefore poses as an interesting field laboratory for exploring prejudices and stereotypes about immigrants and rape attitudes. Based on the study by Aosved and Long (2006) which found positive relationships between rape myth acceptance and several other isms, as well as the studies by George and Martinez (2002) and Katz et al. (2017) which demonstrated a positive association between modern racism and rape victim blame, we hypothesized that modern racism would have a positive relationship with victim blame and a negative relationship with perpetrator blame, as well as a negative relationship with rape perception (the extent to which the event was perceived as a rape).

## Design and statistical analysis

To answer these hypotheses, a survey design methodology was used in the current study. The data were collected with several quantitative measures that were then subjected to statistical analysis. Specifically, the present study investigated the relationship between modern racism and rape victim and perpetrator blame, as well as rape perception (the extent to which the incident was perceived as a rape) using the ordinary least squares regression and multivariate analysis of variance. In other words, we were primarily interested in looking at the relationship between the different variables, and not in establishing causal inferences. An a-posteriori power calculation, with minimum power = .80 (as suggested by Cohen, 1988) and α -level .05, found that 191 participants per sample were needed to find a small to medium ( ρ = 0.2) correlation in the population (Faul et al., 2007).

## Method

### Participants

The participants came from both a community (*n* = 211; 99 women & 112 men) and a student population (*n* = 200; 102 women & 98 men). The majority of participants sampled from the community population had a native Swedish background (114 participants) vs. an immigrant background (97 participants). Their ages ranged from 18 to 84 years (*M* = 30.86, *SD* = 16.25). The majority of the student participants also had a native Swedish background (135 participants) vs. an immigrant background (65 participants). Their ages ranged from 18 to 40 years (*M* = 22.66, *SD* = 3.57).^1^ The community participants were recruited in public spaces such as train and bus stations in southern Sweden, while the student participants were recruited at university campuses in the same region. The proportion of participants with an immigrant background in the current study was higher than the Swedish average, which likely was a consequence of the urban areas of Southern Sweden being more demographically diverse than the rest of Sweden (Statistics Sweden, 2020).

### Materials and measures

#### Rape vignette

Similar to Sjöberg and Sarwar (2020) and Strömwall et al. (2014), the rape vignette used was presented as a short newspaper article (see the Appendix). The article described that Sarah had met an attractive, previously unknown, man named Aron on her way home from a party. When Sarah had arrived home, she had invited Aron into her apartment but changed her mind when the two of them had started kissing inside her apartment. However, Aron forced himself onto Sarah and they completed intercourse. Purposefully, the word rape was excluded from the newspaper article to avoid priming participants before they answered the subsequent questions (Davies & Rogers, 2006; Strömwall et al., 2014). The immigration status of the victim and perpetrator was communicated by explicitly stating in the newspaper article that they either had a native Swedish or immigrant background. No mentions were made of the physical appearances of Sarah or Aron.

#### Rape victim and perpetrator blame items

Following the vignette were four victim blame and four perpetrator blame items which were borrowed from Strömwall et al. (2014). Participants were asked to rate, on a scale from 0% to 100%, to what extent Sarah and Aron could be blamed for the event, respectively. The words victim or perpetrator were eliminated to avoid a potential bias in the participant’s ratings.

#### Rape perception

Participants rated the extent to which they agreed with the following item “To what extent do you perceive the incident in the article as rape?” on a scale from 0% to 100%. Answering 0% meant they did not at all perceive the incident as rape while answering 100% meant they completely perceived the incident as rape.

#### Modern racism

There are different ways of measuring racism (see Henry & Sears, 2002, for a review). This study used a translated version of the Modern Racism Scale (translated into Swedish by Akrami et al., 2000; McConahay, 1983, 1986). The scale measures subtle forms of racism towards immigrants (note the difference from the American scale which measures subtle racism towards *African Americans*). The translated version has been demonstrated to have high internal consistency (Cronbach’s *α* = .82; Akrami et al., 2000). The translated scale is a 9-item measure where responses are given on a 5-point scale anchored by 1 (*strongly disagree*) and 5 (*strongly agree*). One example of an item is “Discrimination of immigrants is no longer a problem in Sweden”. The items of the scale showed acceptable internal consistency (Cronbach’s *α* = .79). Before using the scales, the original authors were contacted and their permission to use the scales was obtained.

#### Demographic information

Before completing the study, participants were asked to provide information about their gender, age, and immigration status. Immigration status was decided based on where the participant’s parents were born. When at least one parent was born outside of Sweden, the participant was deemed to have an immigrant background in line with Sjöberg and Sarwar (2020), and the historical way immigration status was determined by Statistics Sweden (2002).

### Procedure

Participants were approached in public spaces such as train stations and university campuses in the south of Sweden and asked to participate in a short study. They were informed about the potentially sensitive topic of the study (i.e., a study about crime and blaming attitudes) and promised of their anonymity and confidentiality. Fewer than 30% of the approached participants refused to participate while those who wanted to participate gave both oral and written consent. When participants had completed the survey, they were provided with a debriefing page, after which the experimenter thanked them for their participation and debriefed them orally.

## Results

Before the data were subjected to statistical analysis, the negative impact of outliers was reduced by changing them to the next highest/lowest score to keep their ordinal ranking and missing values were imported with the expectation maximization method, in line with Tabachnick and Fidell (2007). Although initial exploration of the data suggested some violations of the assumptions underlying the statistical tests, such as normality, Schmidt and Finan (2018) showed that regression is fairly robust to smaller violations of the assumption of normality, at least for larger samples (such as in this study where the number of observations per variable was >10). Table 1 shows descriptive statistics for the modern racism scale, the victim and perpetrator blame scales, and the rape perception item, respectively.

**Table 1.** Means, standard deviations, minimum, and maximum scores for all the included scales and rape perception.

Scales and item	Mean (SD)	Minimum	Maximum
Modern racism	2.1128^a^ (.6540)	1	4.67
Victim blame	24.2336^b^ (26.5580)	0	100
Perpetrator blame	78.3275^b^ (24.4817)	10	100
Rape perception	77.25^b^ (30.518)	3	100

*Note.* The mean scores for each scale and item were reduced to their respective response scales.

a Possible range, 1–5, ^b^Possible range, 0–100.

As shown in Table 1, the victim, perpetrator blame, and rape perception scales all reached their maximum point of the scale. Only the victim blame and modern racism scales reached their absolute minimum score of their respective scales.

### Modern racism, rape blame, and rape perception

In order to measure the relationship between modern racism and victim and perpetrator blame, and rape perception, the ordinary least squares linear regression analysis was used (which is congruent with the Pearson’s product moment correlation in the singular case; Field, 2012). As demonstrated in Table 2, there was a significant and moderately strong (Cohen, 1988) positive correlation between modern racism and victim blame, and a small to moderate significant negative correlation between modern racism and perpetrator blame and rape perception, respectively.

**Table 2.** Correlation matrix for the victim blame, perpetrator blame, rape perception, and modern racism scales.

Variables	Victim blame	Perpetrator blame	Rape perception
Victim blame	1.00	–	–
Perpetrator blame	−.640***	1.00	–
Rape perception	−.635***	.769***	1.00
Modern racism	.347***	−.274***	−.294***

***p < .001.

In order to determine the explained variance of modern racism on the rape blame and perception measures, three standard multiple regression analyses were carried out with victim blame, perpetrator blame, and rape perception used as dependent variables. Regression analyses performed separately for the student and the community samples did not indicate large differences in the underlying outcomes. Hence, the regression analyses include the whole sample. Bonferroni corrections were employed to avoid an inflated Type I error (corrected α-level = .05/3 ≈ .017). The first model with victim blame as the outcome variable and modern racism as the predictor variable explained 12.1% of the variance in victim blame, *F*(1, 410) = 56.13, *p* < .001. The second model with modern racism as the predictor variable and perpetrator blame as the outcome variable explained 7.5% of the variance in perpetrator blame, *F*(1, 410) = 33.30, *p* < .001. Finally, the last model with modern racism as the predictor variable and rape perception as the outcome variable explained 8.7% of the variance in rape perceptions, *F*(1, 410) = 38.78, *p* < .001.

### Students vs. community members

The study by Sjöberg and Sarwar (2020) failed to explicitly compare students vs. community members’ victim blame, perpetrator blame, and rape perceptions since they were merely used as covariates. In order to investigate if there were any differences between students and community members regarding their level of these variables, a multivariate analysis of variance was conducted (MANOVA). Preliminary analyses demonstrated that all three dependent variables were highly correlated (>.6), which is recommended for MANOVA (Pallant, 2005). Although the Box’s M-test for homogeneity of covariance matrices was significant, $\chi^{2}\left(18\right)=77.982,\;p<.001$ , Tabachnick and Fidell (2007, p. 86) cautioned that for large samples (such as in this study), the Box’s M-test tends to be too strict. Still, for the main analysis, the Pillai’s Trace statistic was reported as it tends to be the most robust (Pallant, 2005; Tabachnick & Fidell, 2007, p. 269).

There was a significant difference between students and community members on the combined dependent variables, *F*(3, 407) = 9.883, *p* < .001, Pillai’s Trace = .068. Looking at the dependent variables separately, and using a Bonferroni adjusted alpha level (adjusted α-level = .05/3 ≈ .017), it was found that the students (*M* = 69.31, *SD* = 86.95) assigned significantly less victim blame compared to the community members (*M* = 123.12, *SD* = 115.99; *F*(1, 409) = 28.082, *p* < .001, $\eta_{p}^{2}=.064$ (approximately a medium effect size; Cohen, 1988). The students (*M* = 81.22, *SD* = 26.91) also perceived the incident significantly more as a rape compared to the community members (*M* = 73.49, *SD =* 33.21; *F*(1, 409) = 6.681, *p* = .010, $\eta_{p}^{2}=.01607$ (approximately a small effect size; Cohen, 1988). However, there was no significant difference in perpetrator blame between the students (*M* = 325.02, *SD* = 89.45) and the community members (*M* = 302.21, *SD* = 104.34; *F*(1, 409) = 5.632, *p* = .018, $\eta_{p}^{2}=.01359$ .

## Discussion

The aim of the current study was to investigate the relationship between modern racism and victim and perpetrator blame, as well as rape perception (the extent to which the incident was perceived as a rape). Based on prior research (e.g., Aosved & Long, 2006; George & Martinez, 2002), we hypothesized that there would be a positive relationship between modern racism and rape victim blame, such that individuals with high levels of modern racism would give the victim higher blame for what happened in the described event. In line with these earlier research findings, this hypothesis was supported. This gives further indications that racism seems to go together with, not only rape myths as indicated by Aosved and Long (2006), but also with rape victim blame (similar to George & Martinez, 2002).

Second, the hypothesized negative relationship between modern racism and rape perpetrator blame was also supported and in line with the above reasoning. This suggests that participants with higher levels of modern racism tended to assign less blame to the perpetrator. In other words, it seems like modern racism is moderately related to both victim (positively) and perpetrator blame (negatively).

Finally, there was also a significant negative relationship between modern racism and rape perception (the extent to which the participant perceived the incident as a rape). In summary, these results suggest that modern racism has a small to moderately strong (Cohen, 1988) relationship with rape victim and perpetrator blaming attitudes as well as rape perceptions. It could be argued that rape victim blame constitutes a subtle form of sexism, especially for ambiguous sexual encounters where it is not always clear that the event was rape. In line with this reasoning, George and Martinez (2002) postulated that there might be an overlap between racism and traditional sex roles, as well as general rape attitudes, suggesting an underlying connection between the different constructs.

As was discussed above, the link between racism and sexism has been suggested by several scholars (Glick & Fiske, 1996; Lewis, 1977; Swim et al., 1995). Hence, one could theorize that there exists an underlying psychological construct which is related to both racial and sexual prejudices. For example, as mentioned previously, there have been some attempts at explaining this link with theories such as the social dominance theory (Sidanius & Pratto, 1999) and, even further back, the authoritarian personality (Adorno et al., 1950). Recent findings have suggested that also other psychological concepts, such as a low need for cognition and political conservatism (which might be related to rigid thinking; Zmigrod, 2020) could affect rape victim blaming (Mancini & Pickett, 2017; Sussenbach et al., 2017). Further research should focus on teasing out the most relevant explanatory links in this context.

### Practical implications for the criminal justice system

The fact that modern racism and victim and perpetrator blame seemed to go together warrants attention for the criminal justice system. It is well established that victims of rape and sexual assault often are not believed fully by the police and, as a result, could experience revictimization (Maier, 2008). Moreover, if judges tend to blame rape victims, there might be a risk that the perpetrators, either get away with their crime, or get a mild sentence. This, in turn, could encourage them and others to engage in similar behaviors in the future. Based on the current findings, it could be theorized that one (not the only) explanation behind this victim blaming could be moderate levels of modern racism in certain legal authorities within the criminal justice system. A first step could therefore be to include measures like the modern racism scale in the recruitment process for policemen and legal professionals (just to name a few). Gathering such information may give a first indication that someone could have a tendency to blame victims of ambiguous sexual crimes more, such as in rape cases, compared to someone with a lower modern racism score.

### Limitations

The first important limitation of this study concerns the methodological design. Since it was correlational, it means that it was not possible to infer causality based on our findings and one therefore has to be very careful in extracting the results of the study to other contexts. A second limitation concerns the sensitive topics that were explored. It is well known that questions about racism and sexism are considered sensitive topics, and therefore, could sometimes be incorrectly reported by research participants (Tourangeau & Yan, 2007). This means that there is a possibility of participants not answering truthfully to the asked questions. Still, it could be hypothesized that this limitation actually attenuated rather than overestimated the relationship between modern racism and victim blaming, and hence, the true relationship might be stronger rather than weaker in the wider population. A final limitation relates to the fact that the specific ethnic background of the participants was not asked for, but simply whether they had an immigrant background or not. This means that anyone from outside of Sweden would be considered to have an immigrant background (irrespective of where they came from), and hence, immigration status represents a somewhat blunt and imprecise measure in this study.

### Future research

Again, since the design of the study was correlational, it was not possible to infer causation based on our findings. Naturally, the next step would be to isolate modern racism and investigate its influence on rape victim and perpetrator blaming attitudes, as well as other “isms”. For example, one could potentially prime participants experimentally with modern racism and look at the effects this could have on rape blaming and sexism in general. It would also be interesting to correlate modern racism with the new political and social divide between globalism and localism, which has been suggested by some scholars to be the defining debate of the new millennium (Williams, 2002). Lastly, one could also conduct more applied research with lawyers and the criminal justice system to get a better understanding of how modern racism plays a role in sentencing decisions of sexual offenders.

## Conclusion

This study showed a low to moderate correlation between modern racism and rape victim and perpetrator blame, as well as rape perception among students and community members in Sweden. This gives further support for the link between racial attitudes and subtle sexism and victim blaming (Aosved & Long, 2006; George & Martinez, 2002; Katz et al., 2017). By investigating the relationship between modern racism and rape victim and perpetrator blaming attitudes in a Northern European context, the existing research was expanded. However, more research still needs to be done to establish causal relationships between modern racism and rape victim blaming attitudes. The coming years will hopefully see an increase in research about this timely topic and also applications to other areas of study so that the understanding of xenophobic and racial attitudes’ relationship with subtle sexist attitudes will be better understood.

## Appendix

The Rape Vignette (adopted from Strömwall et al., 2014)

During a late night last week, Sarah, who has a Swedish/immigrant background, was on her way home from a party in central Mora (a city in Sweden). She was heavily intoxicated. A few blocks away from her flat, she met an attractive unknown man who had been to a different party. Sarah took interest in the man and voluntarily invited him back into her flat to spend the night together. She changed her mind when they had already started kissing in the flat, but the man did not listen and completed coitus against her will. Sarah reported the incident to the police the next morning. There were no witnesses of the event, but with the help of Sarah’s description, the assailant is now identified as Aron and has been arrested by the police. Aron has a Swedish/immigrant background but has never before been charged with violent crimes. He has a university degree and is currently unemployed. During the police interrogation, it was discovered that Aron believed that Sarah consented fully to the act.

## Note

1 The sample was also used in Sjöberg and Sarwar (2020) to investigate related, but different hypotheses. It was collected for Sjöberg’s master’s thesis. According to the International Committee of Medical Journal Editors (2020, March 14), using the same or similar samples from a previous manuscript may be justifiable when the new manuscript adds substantially to the previous manuscript. The study was approved by the Department of Psychology at Lund University in accordance with the Swedish law on ethics for research on human participants.
